# Analysis of Microplastics Released from Plain Woven Classified by Yarn Types during Washing and Drying

**DOI:** 10.3390/polym13172988

**Published:** 2021-09-03

**Authors:** Sola Choi, Miyeon Kwon, Myung-Ja Park, Juhea Kim

**Affiliations:** 1 Department of Material & Component Convergence R&D, Korea Institute of Industrial Technology, Ansan 15588, Korea; solacs@kitech.re.kr (S.C.); mykwon@kitech.re.kr (M.K.); 2 Human Tech Convergence Program, Department of Clothing and Textiles, Hanyang University, Seoul 04763, Korea

**Keywords:** microplastics, microplastic fiber, washing textile, drying textile, polyester yarn types

## Abstract

Microplastics reach the aquatic environment through wastewater. Larger debris is removed in sewage treatment plants, but filters are not explicitly designed to retain sewage sludge’s microplastic or terrestrial soils. Therefore, the effective quantification of filtration system to mitigate microplastics is needed. To mitigate microplastics, various devices have been designed, and the removal efficiency of devices was compared. However, this study focused on identifying different fabrics that shed fewer microplastics. Therefore, in this study, fabric-specific analyses of microplastics of three different fabrics during washing and drying processes were studied. Also, the change in the generation of microplastics for each washing process of standard washing was investigated. The amount of microplastics released according to the washing process was analyzed, and the collected microplastics’ weight, length, and diameter were measured and recorded. According to the different types of yarn, the amount of microplastic fibers produced during washing and drying varied. As the washing processes proceed, the amount of microplastics gradually decreased. The minimum length (>40 µm) of micro-plastics generated were in plain-woven fabric. These results will be helpful to mitigate microplastics in the production of textiles and in selecting built-in filters, and focusing on the strict control of other parameters will be useful for the development of textile-based filters, such as washing bags.

## 1. Introduction

The use and production of plastics have rapidly increased globally since the 1960s; the amount of production rose from 2 million tons in 1950 to 380 million tons in 2015 [1]. Plastic waste is fragmented through various processes, and these fragments have become a cause of marine pollution. In general, microplastic refers to a material composed of small or fine solid particles made of synthetic polymers smaller than 5 mm [2]. On the basis of their origin, microplastics are further defined as “primary microplastics” if they are intentionally produced either for direct use or as precursors to other products, and “secondary microplastics” if they are formed in the environment from the breakdown of larger plastic material [3,4].

Microplastics are used in various consumer, professional, agricultural, and industrial products such as cosmetics, detergents, fabric softeners, maintenance products, paints, coatings, inks, chemicals, construction, pharmaceuticals, and food supplements [2]. They reach the aquatic environment through the wastewater, which is a large conduit [5]. Larger debris is removed in sewage treatment plants, but filters are not explicitly designed to retain microplastic and terrestrial soils that contain microplastic fibers [6]. Therefore, the effective quantification of a filtration system to mitigate microplastics is needed for preventing plastic waste from entering large bodies of water.

The production of 65 million tons of synthetic fiber in 2016 suggests that the massive production of synthetic textiles is the driving cause of microplastic fibers in the environment [6,7,8]. According to the 2020 report from the European Chemicals Agency [2], fiber-shaped microplastics are 3 nm or more, but less than 15 mm in length, with a length to diameter ratio exceeding 3. Natural polymers and biodegradable polymers are excluded [2]. A significant amount of fibers (260–320 × 103 particles/m^3^) was found in the Seine River [9], and after examining microplastics of less than 1 mm in the discharged from a sewage treatment plant located in Jinhae-gu, Changwon-si, in 2013, it was found that 26% of the fibers met the criteria for microplastics (867 ± 470 particles/m^3^) [10]. When fiber-shaped microplastics are absorbed into the body of microorganisms, there is a risk of eating disorders caused by the wrapping of the intestines, thereby hampering growth and reproduction [11]. When PET microfibers of 62–1400 μm in length were exposed to Daphnia magna at a concentration of 12.5–100 mg/L for 48 h, the lethality due to ingestion increased [6]. Therefore, it is necessary to change the perception of collection, reduce the production of microplastics that pose a risk to the environment and human health, and analyze the microplastics emitted from fibers and derive collection methods.

A study that directly estimates the release of microplastics from laundry was first attempted by Browne et al. in 2011 [6]. Starting with Folkö’s research in 2015, various studies on microplastics were produced during laundry focused on microplastics during the laundry [12]. The fabrics almost used were polyester-based [12,13]. In recent studies, fabrics were clearly classified by chemical composition, yarn types, and fabric construction [14,15,16,17,18].

The experiment was conducted on an experimental Launder-O-meter, a laboratory instrument used for conducting accelerated laundering, which uses a small sample size. The Launder-O-meter differs in physical force and movement of fabric compared to that of a large load laundry machine used for home laundry [19,20,21]. Zambrano et al. (2019) used a Launder-O-meter and a large load laundry machine to compare the amount of microplastics released. The Launder-O-meter created approximately 40 times more microplastics than the large load laundry machine. This result was caused by the mechanical action of the stainless-steel balls [20]. Therefore, the use of a large load laundry machine, used for home laundry, was recommended for studies. It is also necessary to measure the microplastics produced by various parameters, such as the individual washing processes, including washing, rinsing, and drying.

Various devices have been designed to divert and capture released microfibers to mitigate the release of microplastics in wastewater effluent. Several studies previously compared the removal efficiency of drum washing machines based on weight [21,22]. However, this study focused on identifying different fabrics that shed fewer microplastics. Focusing on fabrics while strictly controlling other parameters will be useful for developing textile-based filters, such as washing bags. The direction of twist, the number of twists, the thickness and length of the yarn affect the properties of yarn and textiles [23]. Thus, the primary aim of our study was to investigate the release of microfibers according to different yarn types. In addition, other textile parameters, such as chemical composition and fabric construction, were controlled. Three different yarn types with the same chemical composition and fabric construction were selected. Several processes were developed. A secondary aim was to detect microplastics that were reattached to the fabric during the drying process. Finally, to observe the change in the generation of microplastics for each washing process, the entire wastewater effluent was collected separately after each washing process and filtered.

## 2. Experimental

### 2.1. Materials

Three different yarn-type samples with the same chemical composition and weaving method were selected as specimens. Three different fabrics with two types of filament yarns (hard twist yarn, non-twist yarn) and one spun yarn of plain woven polyester were used. The filament hard twist yarn was purchased from Hwan Tex (Korea), the filament non-twist yarn from Yoomyung (Korea), and the spun yarn from Basic (Korea). Cutting edges of the specimen were overlocked using white polyester yarn to prevent thread loosening from the sample. The fabric density of the specimen was measured according to ISO 7211-2, and the weight (ISO 3801), thickness (ISO 4603), and size of the specimens are shown (Table 1). Since the physical forces during washing vary depending on the fabric weight, the fabric weight of the fabric to be tested was set at 500 g [24]. After preparing each specimen, the experiment was performed using five pieces, each weighing 100 g. Since it was challenging to find information about the raw resin of the polyester used in commercially available fabrics, to confirm the fabric composition and construction Fourier Transform Infrared (FTIR, Vertex 80v, Bruker) was used to determine the fabric composition, and a scanning electron microscope (SU8010, HITACHI) was used to confirm the fabric structure and yarn shape.

### 2.2. Washing and Drying

The drum-type washing machine (F9WK, LG electronics, Seoul, Korea) with a capacity of 9 kg was used, and washing was carried out through a standard course without a dummy load. One cycle of the standard washing course is comprised of one laundering and three rinsing processes. The temperature was 40 ± 2 °C of the laundering process, and 20 ± 2 °C in each of the rinsing processes. Washing was performed for a total of 1 h and 20 min, with 40 min for laundering, 10 min for rinsing 1, 10 min for rinsing 2, and 20 min for rinsing 3. Washing water from each cycle was separately collected to determine the amount of microplastics generated during each process. Tap water was used for the washing water and no detergent was added. After the experiment was completed, the washing machine was run empty three times to eliminate microplastic residue or other contaminants in the washing machine. The same experiment was repeated three times.

The dryer used was a dual inverter heat pump type drum dryer (RH9WGN, LG Elec-tronics, Seoul, Korea) with a capacity of 9 kg. Washed fabrics were dried in a standard drying course, with a drying temperature of about 60 °C for 1 h and 40 min. After drying, the drying machine was run three times with no fabric to ensure that no residue was left and the experiment was repeated three times.

### 2.3. Filtration

The wastewater discharged during each cycle was collected in four 20 L containers and was filtered separately. The washing effluents were filtered by means of a peristaltic pump connected with Teflon tubes; throughout quantitative filter papers consisting of cellulose fibers, with a pore size of 5 µm and a diameter of 185 mm (Grade 30, Hyundai micro, Seoul, Korea) were utilized. The filter pore size smaller than the fiber diameter was chosen. The filter paper was placed on a Buchner funnel and with a 5 L filtering flask. The washing water stored in a 20 L container was filtered using a manual liquid pump, and after filtering all water, the container was rinsed with 1 L of tap water to re-move all remaining fibers. The 1 L of tap water was also filtered. The used filtering flask, Buchner funnel, and manual transfer pump were washed with 6 L of tap water before each of the subsequent experiments.

### 2.4. Analysis of Microplastics

Methodologies in previous studies have assessed a select area of filter paper and scaled the count for all fibers from the specimen. However, manual counting of fibers leaves considerable potential for counting errors since fibrous forms, especially micro-plastics during washing, are intertwined across a 3-dimensional spaghetti-like structure [25]. Tiffin et al. (2021) discussed a homogenous distribution of fibers across the entire filter area. Thus, this study quantified microplastic released by weight. The collected microplastics were compared by weight, length, and diameter measurements. The microplastics’ weight was measured using a precision balance after drying with filter paper at a temperature of 26 ± 2 °C and relative humidity of 20% for 24 h. The microplastics generated after drying were measured by comparing the built-in filter’s weight inside the dryer before and after drying. The filter paper’s weight before and after filtering the washing water was calculated according to the ppm equation and com-pared. The mass of collected materials per the mass of the textile, ppm (mg/kg) is calculated by the Equation (1):

ppm (mg/kg) = (*M*_m_ × 1000)/*M*_kg_ (1)

where

ppm is the mass of the collected microplastics per mass of textile in mg/kg
*M*_m_ is the mass of the collected microplastics during washing and drying in mg
*M*_kg_ is the mass of test specimens in kg

The length identification of the three samples was carried out using a digital microscope (magnification of 40×). The captured fibers were spread evenly on the filter paper, and the fiber lengths and diameters were analyzed using the Image J program (NIH, National Institutes of Health, Bethesda, Maryland, MD, USA).

## 3. Results and Discussion

### 3.1. Fourier Transform Infrared and Scanning Electron Microscopy of Specimens

**Figure 1:**
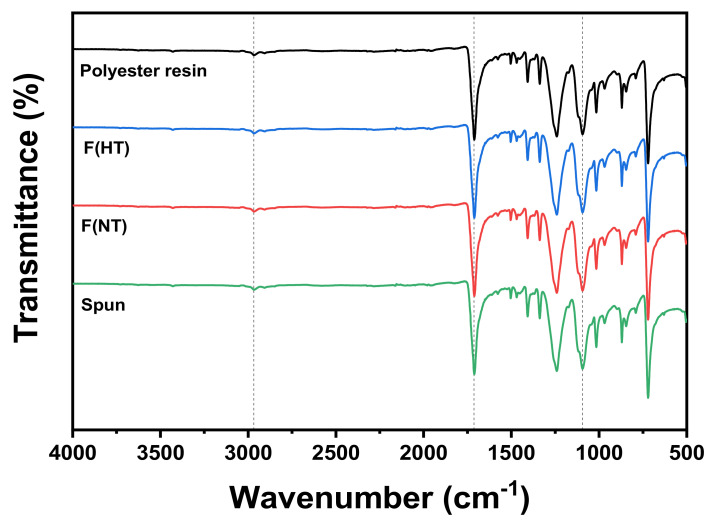
FTIR spectroscopy of polyester resin and three different fabrics classified by yarn types.

Figure 1 shows the FTIR spectrum of the three different fabrics classified by yarn type. All three fabrics exhibited almost the same peak as polyester resin. It can be observed that the unsaturated polyester had a weak band at 2967 cm^−1^, which can be attributed to the C–H elongation [26]. The polyester showed important characteristic absorption in the 1713 cm^−1^ band, which represents the carbonyl group, C=O. The bands close to 740 cm^−1^ represent the elongation of the aromatic nucleus C=C. This occurred due to the presence of the unsaturated double bond (C=C) in the polyester and refers to the vinyl group present in the styrene monomer. The bands close to 1260 and 1117 cm^−1^ occurred due to stretching vibrations C–O–C connected to the aliphatic and aromatic groups, respectively. Through these spectral results, the three samples were confirmed to be polyester, and it was confirmed that the fabric components were chemically similar through FTIR.

**Figure 2:**
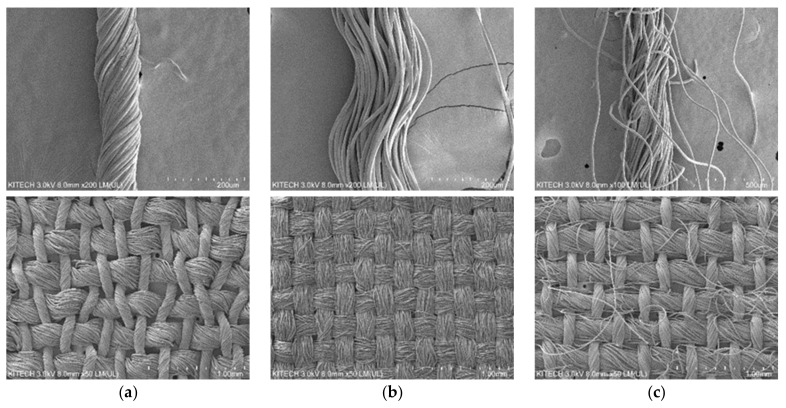
Scanning electron microscope images for identifying yarn type and fabric construction. (**a**) Plain woven with hard twist filament yarn; (**b**) Plain woven with non-twist filament yarn; (**c**) Plain woven with spun yarn.

Also, Figure 2 shows the scanning electron microscopy image of the three different fabrics classified by yarn type. As a result of a scanning electron microscopy, it was confirmed that the types of yarn were different from hard twist filament yarn (Figure 2a), non-twist filament yarn (Figure 2b), and spun yarn (Figure 2c), but all three fabrics were the same as the plain woven. Figure 2a is a hard-twisted filament yarn in which a bundle of fibers is tightly twisted in one direction, compared to Figure 2b is a yarn in which a bundle of fibers is arranged fiber length direction without twisting. Figure 2c is a spun yarn that is composed of a short bundle of fibers that is twisted in one direction more loosely than hard twist yarn. Through these scanning electron microscopy results, the three samples confirmed the same as plain woven and difference in the type of yarn.

At the results of FTIR and SEM, it was confirmed that the three fabrics had similar properties physically and chemically and were only different in yarn type.

### 3.2. Mass of Microplastics Released from Textiles According to Yarn Types during Washing

**Figure 3:**
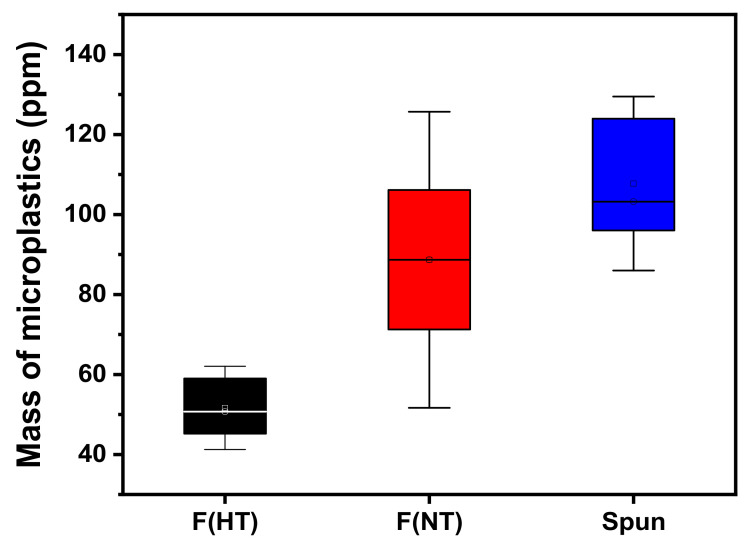
Amount of microplastics according to yarn types during washing.

The results comparing the release of microplastics during washing of plain-woven from hard twist filament yarn, non-twist filament yarn, and spun yarns are shown in Figure 3. The release of microplastics was 51.6 (±6.9) ppm for the hard twist filament yarn, 88.7 (±24.7) ppm for the non-twist filament yarn, and 107.7 (±14.5) ppm for the spun yarn.

The spun yarn showed a higher release of microplastics than the non-twist filament yarn and the hard twist filament yarn. This is because the spun yarn has a higher degree of freedom due to the shorter fiber length than the filament yarn, and the fibers were more easily released from the fabric, resulting in the generation of more microplastics. As a result of comparing the amount of microplastics released by the degree of twist in the filament yarn, non-twist yarn released more microplastics than high-twist yarn. It is considered that the non-twist yarn is more likely to generate microplastics since the friction between the fibers is decreased, resulting in increased fiber freedom.

A similar tendency was observed in Almroth et al. (2018), in which polyester knit with different types of multifilament yarns or spun yarns were used. This research used the counting method inferred from the length measurement results for analyzing the amount of microplastics released [15]. Comparing the overall quantities of microplastics released during washing to those reported by De Falco of 244–296 mg/kg of PES-knit fabric, the release for the present study was lower [17]. Considering the fabric construction, unlike the woven fabric used in this study, knitted fabrics were used in the De Falco et al. (2018). Therefore, it can be expected that fabric construction affected the release of microplastics [4].

As a result of the amount of microplastics produced by the three fabrics with different yarn types, the higher the degree of freedom of the fibers, the higher the amount of microplastics produced. Therefore, to mitigate the release of microplastics, the use of filament yarns, rather than spun yarns, should be applied in synthetic fibers, and a finishing method, such as more twisting or increasing the density of the yarn, is required.

### 3.3. Release of Microplastics as Washing Process

**Figure 4:**
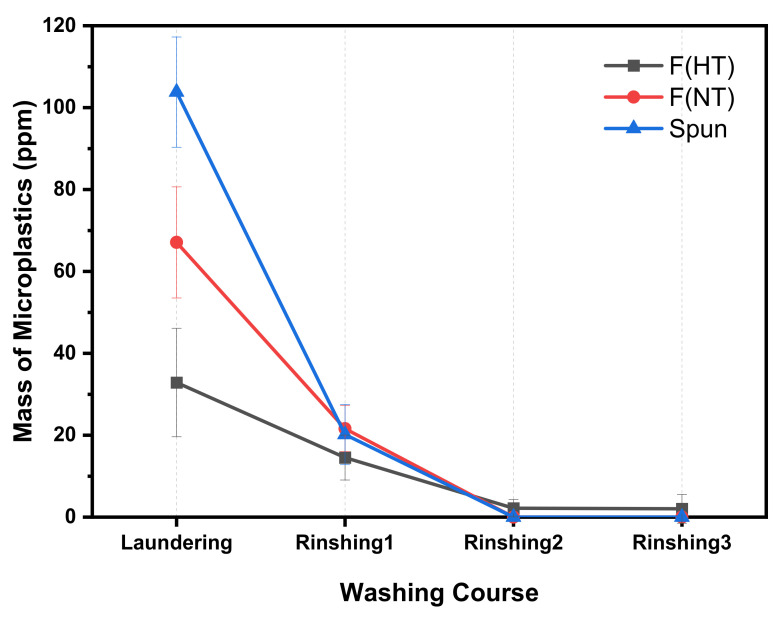
Amount of microplastics generated according to the laundering process.

One cycle of the standard washing course comprises one laundering process and three rinsing processes, and the release of microplastics in each process is shown in Figure 4. Microplastics released in all three fabrics showed the same tendency in the washing process; most microplastics were generated in the laundering process, and the microplastics tended to decrease as the washing proceeded.

Washing was performed for a total of 1 h and 20 min, and the time is longer in the laundering processes (40 min for laundering) than the rinsing processes (10 min for rinsing 1 and 2, and 20 min for rinsing 3). Since the time to receive physical force (washing water, falling off the fabric, and movement of the fabric) is longer in the laundering process than in the rinsing processes, it is understandable that the release of microplastics was released higher in the laundering process. Additionally, since the temperature is higher during laundering (40 °C), the fabrics’ binding forces may be weakened due to the heat energy. Therefore, as the washing process proceeds, the release of microplastics gradually decreases since the time the fabric receives physical force is shortened, and the temperature is lower. In short, the physical forces differ according to each washing process considering temperature and washing time.

### 3.4. Release of Microplastics during Drying

**Figure 5:**
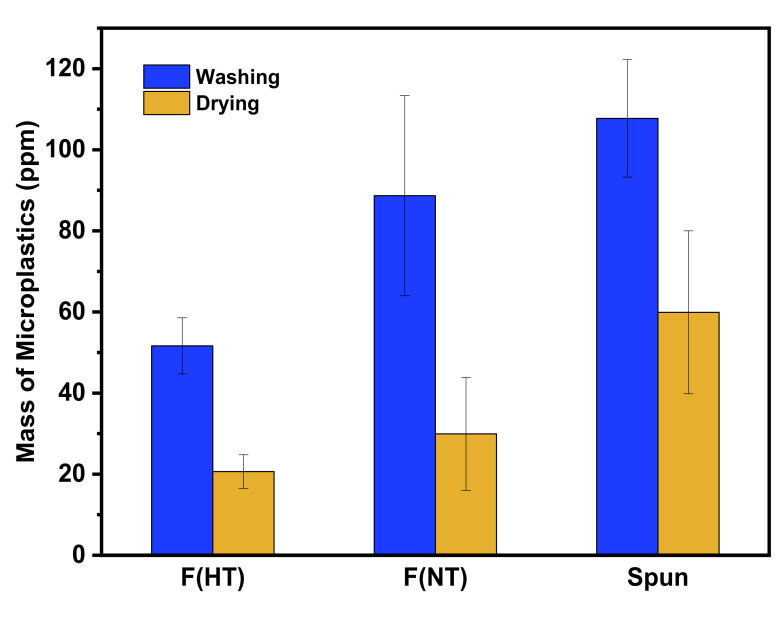
Comparison of microplastics during washing and drying.

Microplastics released during drying were on the order of 59.9 (±20.1) ppm for spun yarn, 29.9 (±13.9) ppm for non-twist filament yarn, 20.6 ppm for hard twist filament yarn, and showed the same tendency as that during washing (Figure 5). As a result of comparing the microplastics released during washing and drying, the amount of microplastics during drying decreased to 66% for spun yarn, 60% for hard twist filament yarn, and 44% for non-twist filament yarn. The amount of microplastics released during the drying process showed the same tendency as the washing process in the order of spun yarn, non-twist filament yarn, and hard twist yarn.

As shown in washing, the same correlation was observed during drying between the degree of freedom of fibers and the release of microplastics depending on the type of yarn. The reason that amount of microplastics during the drying process was reduced compared to washing is as follows: The principal difference between washing and drying processes is the difference in the physical force of water that affects fabrics during washing. Moisture seeping into the fabric during washing makes fabric lose structure, so fibers easily drop out of the fabric. Furthermore, during washing, the fabric receives the physical force of the washing water, the falling of the fabric, and the fabric movement, but only the physical forces of movement and falling of the fabric were present during the drying process. To reduce the production of microplastics, it may be effective to increase the fabric weight to minimize the physical force imparted.

The drying results suggest two things: First, the drying results showed the same tendency as the washing results in the order of spun yarn, non-twist filament yarn, and hard-twist filament yarn, thereby proving our hypothesis again that fabric properties can affect the amount of microfibers. Second, the detachment of microplastics by the drying process is thought to be caused by damage to the fabric due to abrasion during washing. In this case, new microplastics are possibly generated every time the fabric is washed. The results of Napper and Thompson (2016), showed that garments had an initial peak of microplastics shedding in the first 1–4 washes and showed new microplastics are possible to be generated every time the fabric is washed [13]. Although microplastic generation continued to appear after drying, the drying results can be used as an alternative to solve wastewater effluent, and marine pollution as drying does not cause wastewater.

### 3.5. Length of Microplastic Fibers

**Figure 6:**
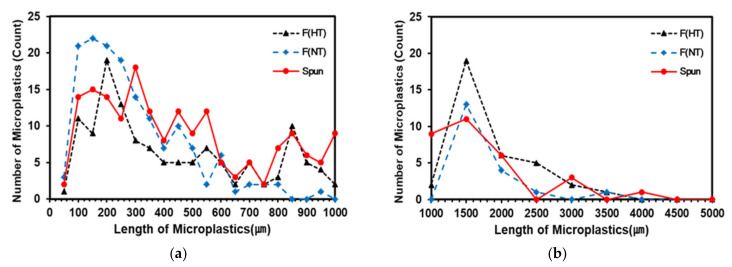
Frequency distribution diagram of microplastics length. (**a**) Length of microplastics from 0 µm to 1000 µm (interval: 100 µm) (**b**) Length of microplastics from 1000 µm to 5000 µm (interval: 500 µm).

The length distribution of fibers released by yarn types showed various length distributions below 1000 µm and a tendency to peak at 1500 µm (Figure 6). As a result of the fiber length distribution from 0 µm to 5000 µm are as follows: the peak points were at 200–300 µm and 1500 µm for hard twist yarn, 100–300 µm and 1500 µm for non-twist filament yarn, and 300–400 µm and 1500 µm for spun yarn. A similar high peak point appeared at 1500 µm in all three samples.

Unlike hard and non-twist filament yarns that showed peaks at 100–300 µm and 1500 µm, slight max peaks appeared at 300–400 µm and 900–1000 µm in spun yarn. Based on the characteristics of the spun yarn, which has lengths of 20–46 mm and is shorter than filament yarn, it is considered that the twists of the spun yarn are easily separated due to swelling and physical force. In contrast, it is believed that the relatively long filament yarns were fragmented after being separated from the fabric.

The diameter used in this study was found to be as small as 10.78 µm, and a smaller filter pore size was used (Table 2). The result of the length analysis of released fibers showed that the minimum lengths were 40.49 µm (FHT), 45.81 µm (FNT), and 44.75 µm (Spun). These are considerably more than 40 µm of microplastics generated in plain-woven fabric. The minimum length (>40 µm) of microplastics is generated in plain woven. Therefore, these results demonstrate that selecting a built-in filter without too small filter pore sizes can mitigate microplastics.

## 4. Conclusions

This study proposes a method to reduce microplastics released from textiles during washing and drying. Focusing on fabrics construction, with strict control of other parameters, will be useful for developing textile-based filters, such as washing bags. The higher the degree of freedom of fibers, the higher the amount of microplastics released, so a finishing method, such as more twisting or increasing the density of the yarn, is required. The results of the washing process can lead to a method for consumers to reduce the amount of microplastics produced in the home. A washing method that shortens the washing time and lowers the temperature is required to reduce physical force. The difference between washing and drying reconfirmed the effect of physical force, and tumble drying is proposed as another alternative for microplastics mitigation. Lastly, fiber length results are expected to contribute as basic data for developing optimal filter pore sizes in washing machine filtration devices to collect microplastics efficiently. To minimize some of the avoidable environmental challenges we currently face, further investigations are needed to develop technology [22]. At the design stage of methods to minimize unintended environmental consequences, we believe that our results are one source for developing effective mitigation tools.

## Figures and Tables

**Table 1:** Characteristics of Specimens.

Specimen		Density		Weight (g/m^2^)	Thickness (mm)	Size (m × m per 100 g)
Yarn Type	Code	Warp (e.p.i ^1^)	Weft (p.p.i ^2^)			
Filament Hard Twist	F(HT)	123.04	88.90	115.0	0.27	1.40 × 0.65
Filament Non Twist	F(NT)	120.09	108.37	85.6	0.18	1.47 × 0.80
Spun	Spun	102.62	84.33	84.4	0.22	1.44 × 0.81

^1^: Ends per inch is the number of warp threads per inch of woven fabric ^2^: Picks per inch is the number of warp threads per inch of woven fabric.

**Table 2:** Descriptive Statistics of Fiber Length and Diameter of Microplastics Released.

Specimen	Length (µm)							Diameter (µm)		
	*n*	*M*	*SD*	*Md*	*SE*	Min.	Max.	*n*	*M*	*SD*
F(HT)	161	648.97	591.54	470.03	46.62	40.49	3108.03	10	14.67	2.20
F(NT)	170	402.40	458.49	240.94	35.16	45.81	3377.19	10	10.78	1.23
Spun	199	572.30	544.88	412.17	38.63	44.75	3679.33	10	12.53	1.94

## Data Availability

Data sharing not applicable.

## References

[1] Geuer R., Jambeck J.R., Law K.R. (2017). Production, use, and fate of all plastics ever made. Sci. Adv..

[2] European Chemicals Agency. https://echa.europa.eu/documents/10162/05bd96e3-b969-0a7c-c6d0-441182893720.

[3] Kershaw P.J. (2015). Sources, fate and effects of microplastics in the marine environment: A global assessment. Reports and Studies GESAMP, Joint Group of Experts on the Scientific Aspects of Marine Environmental Protection.

[4] De Falco F., Cocca M., Avella M., Thompson R.C. (2020). Microfiber release to water, via laundering, and to air, via everyday use: A comparison between polyester clothing with differing textile parameters. Environ. Sci. Technol..

[5] McIlwraith H.K., Lin J., Erdle L.M., Mallos N., Diamond M.L., Rochman C.M. (2019). Capturing microfibers–marketed technologies reduce microfiber emissions from washing machines. Mar. Pollut. Bull..

[6] Browne M.A., Crump P., Niven S.J., Teuten E., Tonkin A., Galloway T., Thompson R. (2011). Accumulation of microplastic on shorelines woldwide: Sources and sinks. Environ. Sci. Technol..

[7] Nonwovens Industry. https://www.nonwovens-industry.com/contents/view_online-exclusives/2017-05-23/the-fiber-year-reports-on-2016-world-fiber-market.

[8] Boucher J., Friot D. (2017). Primary Microplastics in the Oceans: A Global Evaluation of Sources.

[9] Dris R., Gasperi J., Rocher V., Saad M., Renault N., Tassin B. (2015). Microplastic contamination in an urban area: A case study in Greater Paris. Environ. Chem..

[10] Korea Institute of Ocean Science & Technology. http://wwwsciwatch.kiost.ac.kr/kor.dohandle/2020.kiost/36946.

[11] Jemec A., Horvat P., Kunej U., Bele M., Kržan A. (2016). Uptake and effects of microplastic textile fibers on freshwater crustacean Daphnia magna. Environ. Pollut..

[12] Folkö A. (2015). Quantification and Characterization of Fibers Emitted from Common Synthetic Materials during Washinglaundering.

[13] Napper I.E., Thompson R.C. (2016). Release of synthetic microplastic plastic fibres from domestic washinglaundering machines: Effects of fabric type and washinglaundering conditions. Mar. Pollut. Bull..

[14] Yang L., Qiao F., Lei K., Li H., Kang Y., Cui S., An L. (2019). Microfiber release from different fabrics during washinglaundering. Environ. Pollut..

[15] Almroth B.M.C., Ström L.Å., Roslund S., Petersson H., Johansson M., Persson N.K. (2018). Quantifying shedding of synthetic fibers from textiles; a source of microplastics released into the environment. Envrionmental Sci. Pollut..

[16] Cai Y., Yang T., Mitrano D.M., Heuberger M., Hufenus R., Nowack B. (2020). Systematic study of microplastic fiber release from 12 different polyester textiles during washinglaundering. Environ. Science Technol..

[17] De Falco F., Gullo M.P., Gentile G., Di Pace E., Cocca M., Gelabert L., Brouta-Agnésa M., Rovira A., Escudero R., Avella M. (2018). Evaluation of microplastic release caused by textile washing processes of synthetic fabrics. Environ. Pollut..

[18] De Falco F., Di Pace E., Cocca M., Avella M. (2019). The contribution of washing processes of synthetic clothes to microplastic pollution. Sci. Rep..

[19] Hernandez E., Nowack B., Mitrano D.M. (2017). Polyester textiles as a source of microplastics from households: A mechanistic study to understand microfiber release during washing. Environ. Sci. Technol..

[20] Zambrano M.C., Pawlak J.J., Daystar J., Ankeny M., Cheng J.J., Venditti R.A. (2019). Microfibers generated from the laundering of cotton, rayon and polyester based fabrics and their aquatic biodegradation. Mar. Pollut. Bull..

[21] Jönsson C., Levenstam Arturin O., Hanning A.C., Landin R., Holmström E., Roos S. (2018). Microplastics shedding from textiles—Developing analytical method for measurement of shed material representing release during domestic washing. Sustainability.

[22] Napper I.E., Barrett A.C., Thompson R.C. (2020). The efficiency of devices intended to reduce microfibre release during clothes washing. Sci. Total Environ..

[23] Kim S.R. (2000). Clothing and Materials.

[24] Kelly M.R., Lant N.J., Kurr M., Burgess J.G. (2019). Importance of water-volume on the release of microplastic fibers from laundry. Environ. Sci. Technol..

[25] Tiffin L., Hazlehurst A., Sumner M., Taylor M. (2021). Reliable quantification of microplastic release from the domestic laundry of textile fabrics. J. Text. Inst..

[26] Maradini G.D.S., Oliveira M.P., Carreira L.G., Guimarães D., Profeti D., Dias Júnior A.F., Boschetti W.T.N., Oliveira B.F.D., Pereira A.C., Monteiro S.N. (2021). Impact and Tensile Properties of Polyester Nanocomposites Reinforced with Conifer Fiber Cellulose Nanocrystal: A Previous Study Extension. Polymers.

